# *Actinobacillus pleuropneumoniae* Interaction With Swine Endothelial Cells

**DOI:** 10.3389/fvets.2020.569370

**Published:** 2020-10-29

**Authors:** Berenice Plasencia-Muñoz, Francisco J. Avelar-González, Mireya De la Garza, Mario Jacques, Adriana Moreno-Flores, Alma L. Guerrero-Barrera

**Affiliations:** ^1^Laboratorio de Biología Celular y Tisular, Departamento de Morfología, Centro de Ciencias Básicas, Universidad Autónoma de Aguascalientes, Aguascalientes, Mexico; ^2^Laboratorio de Estudios Ambientales, Departamento de Fisiología y Farmacología, Centro de Ciencias Básicas, Universidad Autónoma de Aguascalientes, Aguascalientes, Mexico; ^3^Departamento de Biología Celular, Centro de Investigación y de Estudios Avanzados del IPN, Ciudad de México, Mexico; ^4^Groupe de Recherche sur les Maladies Infectieuses en Production Animale, Faculté de Médecine Vétérinaire, Université de Montréal, Saint-Hyacinthe, QC, Canada

**Keywords:** *Actinobacillus pleuropeumoniae*, cytoskeleton, bacterial cell adhesion, bacterial cell internalization, endothelial cell

## Abstract

*Actinobacillus pleuropneumonia* is a swine (host) specific respiratory pathogen and the etiological agent of swine pleuropneumonia which affects pigs of all ages, many being asymptomatic carriers. This pathogen has high morbidity and mortality rates which generates large economic losses for the pig industry. Actinobacillus pleuropneumoniae is a widely studied bacterium, however its pathogenesis is not yet fully understood. The prevalence of the 18 serotypes of *A. pleuropneumoniae* varies by geographic region, in North American area, more specifically in Mexico, serotypes 1, 3, 5b, and 7 show higher prevalence. *Actinobacillus pleuropneumoniae* is described as a strict extracellular pathogen with tropism for lower respiratory tract. However, this study depicts the ability of these serotypes to adhere to non-phagocytic cells, using an endothelial cell model, as well as their ability to internalize them, proposing it could be considered as an intracellular pathogen.

## Introduction

*Actinobacillus pleuropneumoniae* (App) is a respiratory pathogen member of the family Pasteurellaceae and etiological agent of swine pleuropneumonia (1, 2). Depending on its nicotinamide adenine dinucleotide (NAD) requirements, it is classified into two biotypes, biotype 1 groups NAD-dependent strains, biotype 2 groups NAD-independent strains. Currently 18 different serotypes are recognized. The biotypes and serotypes of *A. pleuropneumoniae* have genetic, structural, and virulence differences. Serotyping is mainly based on capsular antigens. The presence and prevalence of serotypes varies between countries. In North America serotypes 1 and 5 historically are the most commonly isolated. In recent findings most prevalent App serotypes in Canada were 5 and 7 with a notable reduction of serotype 1 incidence due to serological control of breeders. While in Mexico serotype 1 still has a high prevalence alongside serotypes 3, 5, and 7 (3–8).

Swine pleuropneumonia caused by App and is a major problem for swine production. It has a significant impact on animal welfare and economic production, due to high morbidity and mortality rates, the reduction of average daily weight gain and feed conversion rates, loss due to slaughter and intervention costs (2, 9, 10). *Actinobacillus pleuropneumoniae* can be transmitted between herds through various vectors, including direct contact with infected animals, contaminated aerosols and fomites (10, 11).

Antibiotic and vaccination-based therapies help reduce the severity of disease signs and decrease mortality rates. However, they are not effective in eliminating bacteria; even pigs that survive the acute phase can reach subclinical infection and be persistent carriers, harboring App in the tonsils and suffering from chronic lung lesions (12–15).

App virulence is mainly determined by the production of pore forming RTX toxins: ApxI, ApxII, and Apx III. Which have hemolytic and cytotoxic properties and are secreted by the different serotypes in various combinations. ApxI is expressed by serotypes 1, 5, 9, 10, 11, and 14. ApxII is produced by all serotypes except 10 and 14; and ApXIII is present in serotypes 2, 4, 6, 8, and 15 (16). ApxIV is found in all serotypes, is specific to App therefore highly used in diagnostics for species identification (16, 17).

Many other App virulence factors have been described. Such as siderophores, transferrin binding proteins for iron acquisition and binding of porcine hemoglobin by lipopolysaccharide and outer membrane proteins involved in maltose uptake. The lesion induction by Apx toxins, that provokes cellular lysis. Presence of fimbriae, lipopolysaccharides, and adhesins involved in adhesion to name a few (18). Additionally, *A. pleuropneumoniae* can form biofilms that decreases its susceptibility to bactericides and the host's immune system. Likewise, other resistance genes and plasmids have been identified (12–14). Although the pathobiology of App infection has been extensively studied (17, 19), some aspects of its pathogenesis, mainly its relationship with host cells, are still to be known.

To establish infection, pathogenic bacteria must evade the immune response and host defense mechanisms. This is achieved thanks to the interaction established with non-phagocytic cells, such as endothelial and epithelial cells, managing to adhere, internalize, and in some cases survive and replicate (20).

The Gram-negative bacterium *Haemophilus influenzae*, member of *Pasteurellaceae*, colonizes the upper respiratory tract and causes middle ear infection, sinusitis, conjunctivitis, chronic bronchitis, bronchiectasis, cystic fibrosis, and pneumonia. This bacterium adheres to respiratory epithelial mucus. Provokes host cell cytoskeleton rearranges, form lamellipodia which surrounds adherent bacteria and produces a membrane-bound vacuole. Its invasion is inhibited by cytochalasin D (actin polymerization disruptor). Nevertheless, micropinocytosis may be the predominant invasion pathway for this pathogen, in which case, actin cytoskeleton has not this important role (21).

Also in Pasteurellaceae, *Actinobacillus suis*, an opportunistic pig pathogen, colonize tonsils in asymptomatically way. This bacteria has similarities with App, shares several virulence factors and causes similar diseases such as hemorrhagic fibrinopleuropneumonia and septicemia. Even though its pathogenesis is poorly understood, it is known that the OmpA protein, also present in App, plays a key role in adhesion to the tonsils and is crucial for achieving initial colonization and invasion of the central nervous system (22). *Actinobacillus pleuropneumoniae* has been described as an extracellular pathogen capable of adhering to host cell surface as tracheal or alveolar cells, but unable to invade them (6, 23).

The mammalian lung's structure is design for its main function, the gas exchange, which takes place in the alveolar region where air and blood are brought in close proximity over a large surface. Blood flows in a capillary network embedded in inter-alveolar septa. The barrier between air and blood consists of a continuous alveolar epithelium (a mosaic of type I and type II alveolar epithelial cells), a continuous capillary endothelium and the connective tissue layer in-between (24). Endothelial cells lining the pulmonary microvessels are in proximity to the alveolar epithelial cells and their individual basement membranes fused together in order to facilitate gas diffusion. There is evidence that respiratory pathogen as influenzae virus may sometimes spread beyond respiratory tract to cause disseminated infection taken advantage of the proximity between the alveolar epithelium and the endothelia (25, 26). Lubkin and Torres (26) mention that pathogenic bacteria use the bloodstream as a means of transport through the body, so that at some point they come in contact with the endothelium (27). This can exacerbate the lung pathologies (28–32).

Bacterial pathogens that causes systemic infections disseminate through vascular or lymphatic vessels to different target organs. To achieve this, they must adhere to the endothelial tissue, causing the rearrangement of the endothelial membrane through various stimuli, modifying the permeability of the endothelium or using endothelial vasculature or caveolins to reach the bloodstream (33–35).

App is considered a pathogen exclusive of the porcine respiratory system. However, it has been found in tonsils of carrier pigs. Sometimes produces arthritis, osteomyelitis, hepatitis, meningitis, and nephritis, in which the only detectable pathogen is *A. pleuropneumoniae*, it is not known if this represent the possibility of App spread in cases with extensive damage (26, 36). In this sense, the purpose of the present study was to confirm App serotypes 1, 3, 5b, and 7 (4074, JL03, L20, and AP76 strains) adhesion capacity as well to determine *A. pleuropneumoniae* invasion ability into a non-phagocytic cell culture model of endothelial cells as a novel infection mechanism.

## Materials and Methods

### Endothelial Cell Culture

Pig aortic endothelial cell culture previously established by Burciaga-Nava et al. (37) was maintained using Dulbecco's Modified Eagle Medium (DMEM) (Gibco, BRL, Grand Island, NY) supplemented with 10% fetal bovine serum (FBS) (Gibco, BRL, Grand Island, NY), 100 μg/ml penicillin/streptomycin and 2.5 μg/ml amphotericin at 37°C in a humid environment with 5% CO_2_ and 95% air.

### Bacterial Culture Growth

*Actinobacillus pleuropneumoniae* 4074, JL03, L20, and AP76 strains (serotypes 1, 3, 5b, and 7, respectively) were grown in Brain Heart Infusion (BHI) medium (BD BIOXON, Madrid, Spain) supplemented with 15 μg/ml nicotinamide adenine dinucleotide (Sigma Chemical Co., St. Louis, Mo, USA) at 37°C. Overnight bacterial cultures with optical density of 0.6 at 600 nm were harvested by centrifugation (10,000 g × 5 min) and resuspended in DMEM medium until reaching an optical density of 0.6 at 600 nm providing the final concentration required for the assays (6, 38) based on previous growth curve models performed for each strain (data not included).

### Adhesion Assay

Endothelial cells (10^4^) were seeded into 48-well-tissue culture plates. After overnight incubation confluent cells were infected with the different App strains at a multiplicity of infection (MOI) of 10:1. Plates were incubated for 0.5, 1, and 2 h, and subsequently washed several times with Dulbecco's Phosphate-Buffered Saline (DPBS) to remove non-adherent bacteria. Cells with adhered bacteria were released from the wells using Tryple™ (Gibco, BRL, Grand Island, NY) and resuspended in BHI medium. Serial dilutions were performed and plated on agar to determine the number of bacteria adhered to the endothelial cells by colony forming unit (CFU) per well counting (6, 38, 39).

### Internalization Assay

Endothelial cells were seeded into 48-well-culture plates and infected as previously described allowing the interaction for 3 h. After incubation non-adhered bacteria was removed with DPBS washes. Gentamicin protection assay was performed by incubating cultures in DMEM supplemented with 100 μg/ml gentamicin for 1 h at 37°C. Cultures were washed three times with DPBS and cells were lysed with 100 μl pre-chilled sterile distilled water. The lysate underwent several dilutions and was plated on agar plates for subsequent CFU count (6, 40, 41).

Internalization percentage was calculated dividing the organisms inoculated (CFU/ml) by organisms recovered (CFU/ml) and multiplying by 100. Results are presented as mean ± SD (42).

### Immunofluorescence Labeling for Cytoskeleton, Endoplasmic Reticulum, and Golgi Apparatus

To test App invasion, endothelial cells were seeded (10^4^) (37) into permanox Lab-Tek chamber slides (Thermo Scientific) and incubated overnight. Cells were infected with the different App strains with a MOI of 3:1. After a 3-h incubation, non-adherent bacteria were removed by a DPBS wash and subsequently fixed 30 min using formaldehyde 3.7% at room temperature. Then cells were gently wasehed three times with DPBS.

After fixation, endothelial cells' membrane was permeabilized with 0.1% Triton X-100 (US Biological, Salem, Ma, USA) for 5 min at room temperature (43, 44), followed by 2 washes with DPBS. Once the membrane was permeabilized to observe the implication of different cellular organelles in App invasion by immunofluorescent labeling different labels were used according to the organelle to be observed, which are described below. Several repetitions were performed for each organelle.

#### Actin Labeling

After cell fixation and permeabilization, cells were washed twice with DPBS. Then cells were blocked with 1% BSA-DPBS. Cells were washed twice with DPBS and then actin cytoskeleton was labeled with Alexa Fluor 488 Phalloidin [1:100] (Molecular Probes, Eugene, Oregon, USA) for 30 min at 37°C, after labeling cells were gently washed three times with DPBS. Then cells were blocked with 1% BSA-DPBS, washed twice with DPBS and then cells were incubated with primary antibody against App. After that, cells were washed twice with DPBS, cells were incubated with Anti Rabbit IgG Alexa Fluor 594. The cells were washed twice with DPBS, then nuclei were labeling with Hoetch (2 μM) during 15 min at room temperature, then were washed twice, and finally cells were mounted with Prolong Gold (Molecular Probes). Cell control only were labeled with phalloidin as described above.

#### Tubulin Labeling

After cell fixation and permeabilization, cells were washed twice with DPBS. Then cells were blocked with 1% BSA-DPBS. Cells were washed twice with DPBS and then tubulin cytoskeleton was labeled by 2 h incubation at 37°C using a primary monoclonal antibody mouse anti α-tubulin [2 μM] (Sigma Chemical Co., St. Louis, Mo, USA), after this, cells were gently washed twice with DPBS. Then cells were incubated 1 h at 37°C with the secondary monoclonal antibody Alexa Fluor 488 Goat anti-mouse IgG [2 μM] (Sigma Chemical Co., St. Louis, Mo, USA). Then cells were blocked with 1% BSA-DPBS, washed twice with DPBS and then cells were incubated with primary antibody against App. After that cells were washed twice with DPBS, cells were incubated with Anti Rabbit IgG Alexa Fluor 594. The cells were washed twice with DPBS, then nuclei were labeling with Hoechst 33258 [2 μM] (Molecular Probes, Eugene, Oregon, USA) during 15 min at room temperature, then were washed twice, and finally cells were mounted with Prolong Gold (Molecular Probes, Eugene, Oregon, USA). Cell control only were labeled for tubulin as described above.

#### Actin and Tubulin Influence on App Internalization Test

In order to stablish the actin or tubulin cytoskeleton role in App entry to the endothelial cell, cultures underwent a pre-incubation for 5 min with 1 μM actin disruptor Cytochalasin D (CD) (Sigma Chemical Co., St. Louis, Mo, USA) or for tubulin, 1 μM tubulin disruptor Colchicine (Sigma Chemical Co., St. Louis, Mo, USA) (45–47). After the pretreatment with cytoskeleton disruptors, the cells were gently washed twice with DPBS. Then they were labeled with phalloidin or with the anti α-tubulin antibody, as is described above.

#### Golgi and Endoplasmic Reticulum Labeling

Golgi Apparatus was labeled using CellLight Golgi RFP BacMam 2.0 (Molecular Probes, Eugene, Oregon, USA) as for endoplasmic reticulum (ER) SelectFX Alexa Fluor 488 Endoplasmic Reticulum Labeling Kit was used (Molecular Probes, Eugene, Oregon, USA). Labeling was performed following manufacturers' protocols.

*Actinobacillus pleuropneumoniae* was labeled for 2 h at 37°C with a primary polyclonal antibody Rabbit anti-App obtained by Guerrero-Barrera et al. (48) and a secondary polyclonal antibody Alexa Fluor 594 Goat anti-rabbit IgG [2 μM] (Molecular Probes, Eugene, Oregon, USA) for 1 h at 37°C. Samples were observed with a Carl Zeiss LSM700 confocal scanning microscope at 40X.

The length of 40 randomly selected nuclei from samples of the different interactions performed were measured using the blue ZEN software (Carl Zeiss, Germany) to compare the difference between the control and the cells with the internalized App. Results are presented as mean ± SD.

### Vacuole Presence Evaluation by Acidic Organelles Labeling

Using Lab Tek chambers internalization assay was performed as mentioned, with positive control *E. coli* Bl21 (pUCmT::Vat) as Díaz et al. (49) propose. After interaction cultures were washed and incubated for 30 min with fresh DMEM media with Lysotracker Deep Red reagent [75 nm] (Molecular Probes, Eugene, Oregon, USA), after that cells were washed twice with DPBS. Then, samples were fixed and mounted as previously specified.

### *A. pleuropneumoniae* Serine Protease Expression Analysis

Total proteins from App strains 4074, JL03, L20, and AP76, *E. coli* Bl21 (pUCmT::Vat) and *E. coli* Bl21 empty vector (positive and negative control, respectively) (49) were extracted by boiling for 3 min with staining buffer and TLCK. Protein concentration was measured by Bradford technique (50). Samples were electrophoresed on an 10% SDS-PAGE gel and transferred to a nitrocellulose membrane. Membrane was blocked with 5% skim milk (Gibco, BRL, Grand Island, NY), then was washed with PBS-Tween 0.1% three times, and incubated over night at 4°C with primary polyclonal antibody anti Vat SPATE [1:1,000] at 4°C, this used to test the Serine Protease presence. Then the membrane was washed three times with PBS-Tween 0.1% (51), and then was incubated with the secondary antibody anti rabbit IgG-HRP [2:10,000] (Molecular Probes, Eugene, Oregon, USA) for 1 h at room temperature. Finally the membrane was revealed with DAB (Sigma Chemical Co., St. Louis, Mo, USA).

### Statistical Analysis

All experiments were performed by triplicate. The data was analyzed by ANOVA and two-way ANOVA tests for statistical significance at a *P*-value of 0.05 using GraphPad Prism version 7.0 (GraphPad Software, San Diego, CA, USA).

## Results

### Adhesion Assay

**Figure 1:**
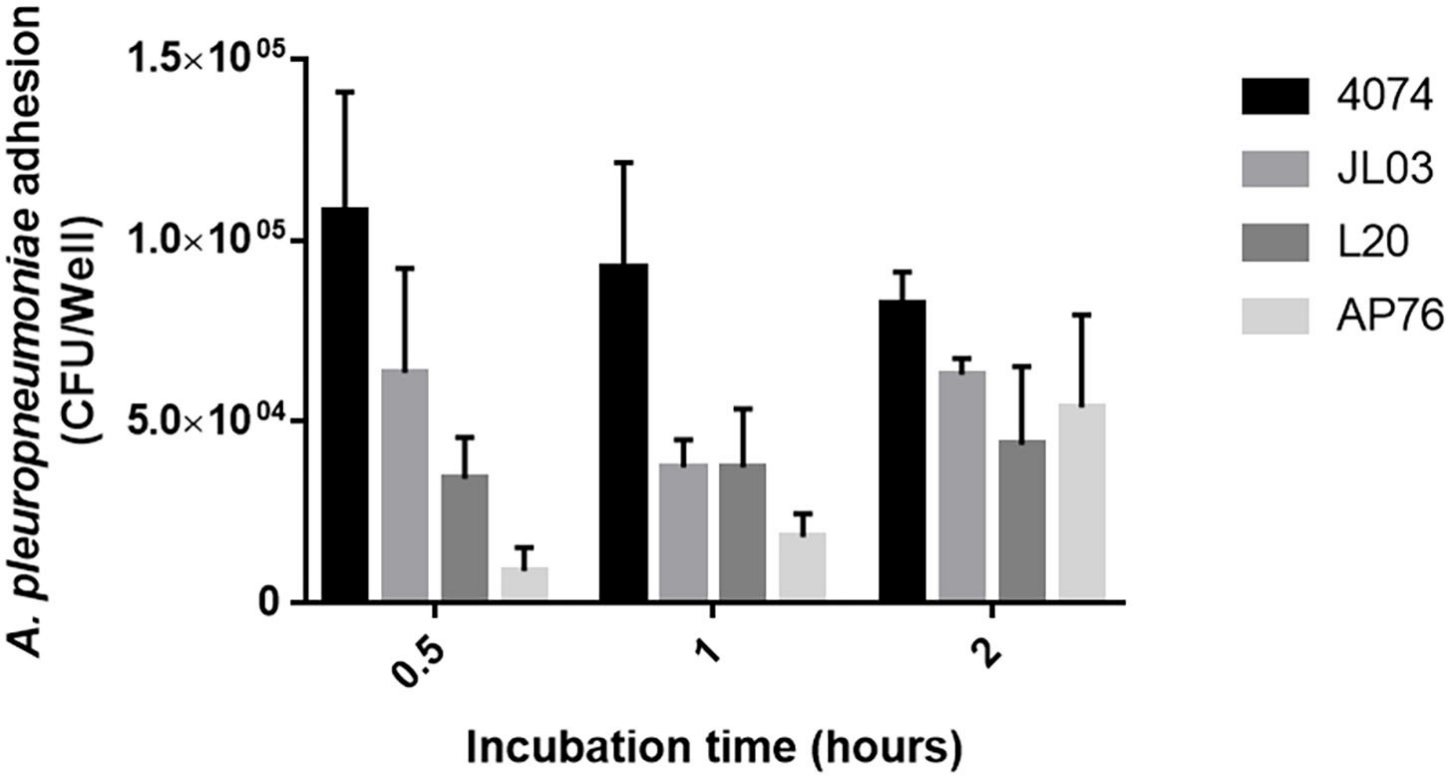
Adherence of *A. pleuropneumoniae* S4074, JL03, L20, and AP76 to swine aortic endothelial cells observed from 0.5 1 and 2 h of incubation. Error bars represent standard error (SE).

*Actinobacillus pleuropneumoniae* 4074, JL03, L20, and AP76 strain adhesion capacity to endothelial cells was determined. App 4074 shows higher adhesion rates compared with the rest of the serotypes studied, however the number of adhered CFUs decreases with increasing incubation period, contrary to L20 and AP76 which increase their adhesion in relation to incubation time; while JL03 strain shows an irregular pattern by decreasing the amount of adhesion at 1 h compared at 0.5 h and increasing again after 2 (Figure 1). Statical analysis data collected for each strain show that the adhesion difference between strains is not significant.

### Internalization Assay and Immunofluorescent Labeling

The invasion capacity of *A. pleuropneumoniae* was investigated. CFU number confirm the ability to enter the host cell of the 4 App strains studied, although the internalization capacity of these strains was relatively low. *A. pleuropneumoniae* JL03 has a higher percentage of invasion (4.6%), followed by L20 (1.75%), AP76 (1.6%), and 4074 with an invasion of 1.3% (Table 1).

**Table 1:** Internalization percentage of *A. pleuropneumoniae* S4074, JL03, L20, and AP76 to swine aortic endothelial cells (MOI 10:1) at 3 h.

**Serotype**	**CFU/Well**	**Internalization percentage**
1	5.50 × 10^3^	1.3 ± 1.15%
3	1.87 × 10^4^	4.6 ± 1.13%
5b	7.03 × 10^3^	1.75 ± 0.69%
7	6.4 × 10^3^	1.6 ± 0.64%

**Figure 2:**
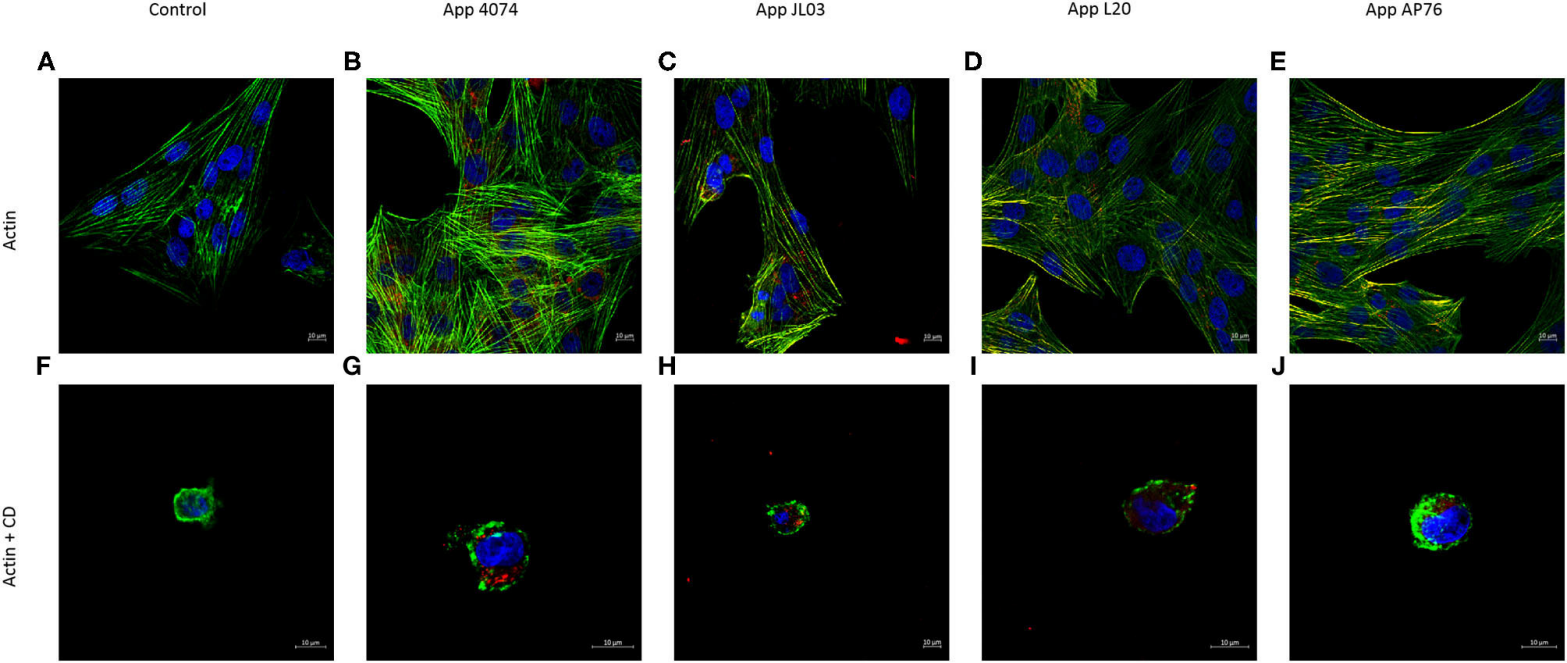
Actin cytoskeleton implication in *A. pleuropneumoniae* internalization to swine aortic endothelial cells after 3 h of incubation. **(A)** Actin cytoskeleton of swine endothelial cells stained in green with Alexa Fluor 488 Phalloidin; **(B–E)** *A. pleuropneumoniae* internalized in swine endothelial cells, labeled with primary polyclonal antibody, and secondary antibody Alexa Fluor 594 (red); **(F)** Actin cytoskeleton incubated with CD; **(G–J)** App internalized and lodge in perinuclear area of swine endothelial cells treated with CD.

Once App's ability to enter the host was observed by CFU counts, the involvement of the actin and tubulin cytoskeleton in the internalization mechanism was analyzed. Figure 2 shows the normal distribution of actin microfilaments inside the endothelial cell, after the incubation of endothelial cells with the 4 App strains, there was no notable changes in actin rearrangement, but the bacteria was observed inside the endothelial cell, some of them housed in the perinuclear area, as well as their co-localization with the actin filaments in some areas. After internalization assay with endothelial cells pre-treated with CD, a well-known actin disruptor, the different App strains were still found inside the host regardless actin cytoskeleton presence.

**Figure 3:**
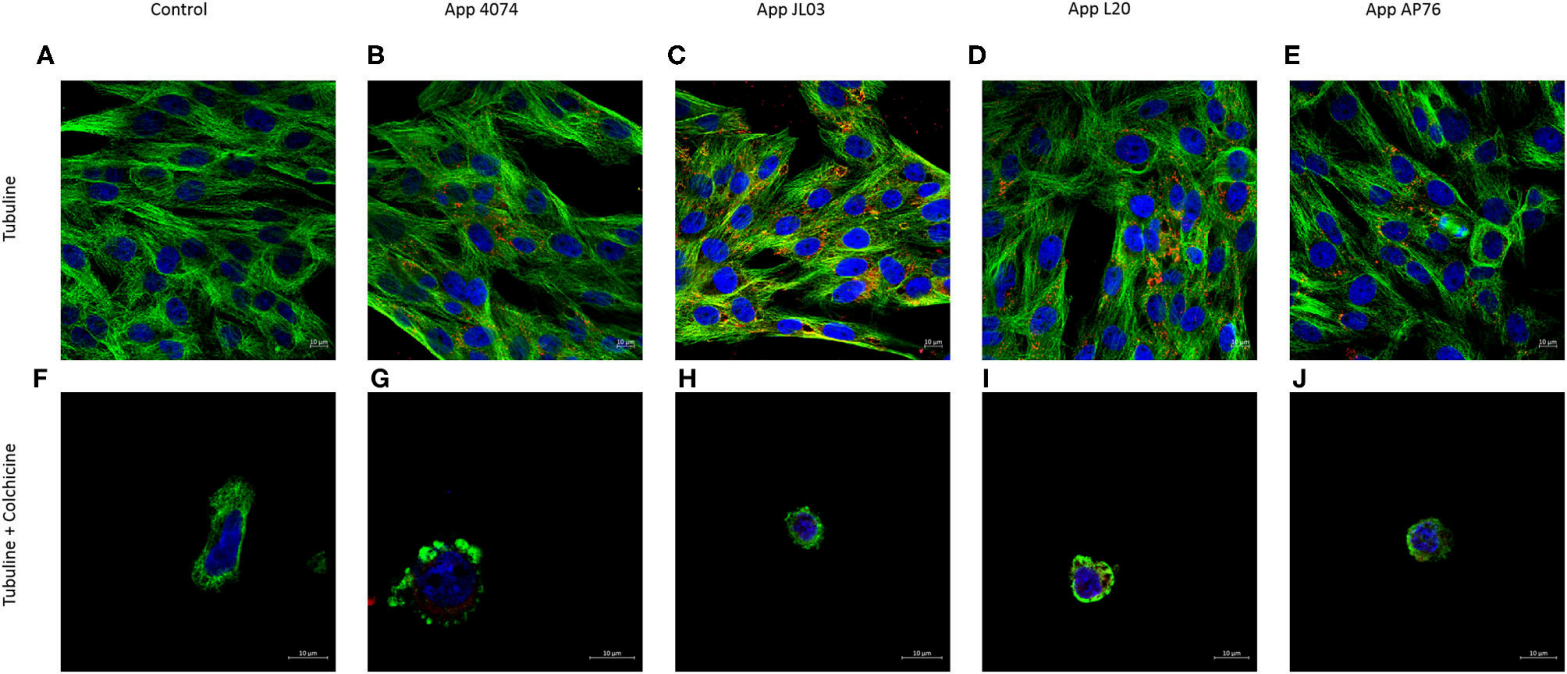
Tubulin cytoskeleton implication in *A. pleuropneumoniae* internalization to swine aortic endothelial cells after 3 h of incubation. **(A)** Tubulin cytoskeleton of swine endothelial cells stained in green with primary monoclonal antibody and secondary monoclonal antibody Alexa Fluor 488; **(B–E)** *A. pleuropneumoniae* internalized in swine endothelial cells labeled with primary polyclonal antibody and secondary antibody Alexa Fluor 594 (red); **(F)** Tubulin cytoskeleton treated with colchicine; **(G–J)** App maintains ability to internalized in endothelial cells treated with colchicine. Nuclei counterstained with Hoechst 33258.

The tubulin cytoskeleton participation in the App internalization is shown in Figure 3. Infected cultures showed the 4 App strains inside the endothelial cells, some of them forming clumps in the perinucleus. Bacteria can also was observed spreaded in the cell, inside round intracytoplasmic zones similar to vesicles, as well as in the circumference of these structures showing co-localization with microtubules in these and other areas. In pre-treated cultures with colchicine (tubulin disruptor) App was also observed inside the cells.

**Figure 4:**
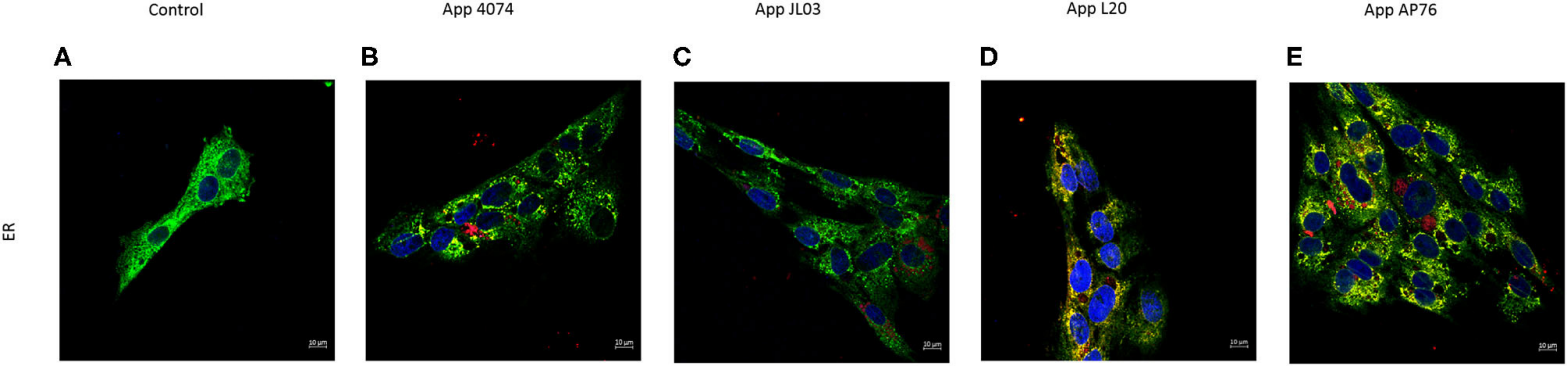
*Actinobacillus pleuropneumoniae* internalization effect on Endoplasmic Reticulum. **(A)** Normal distribution of endoplasmic reticulum stained with SelectFX Alexa Fluor 488 endoplasmic reticulum labeling kit (green); **(B–E)** App (red) internalized into endothelial cells. Co-localization of App and ER is observed however no major ER distribution changes are shown.

The relationship of internalized *A. pleuropneumoniae* with organelles of the endothelial cell involved in the transport of substances was investigated. The labeling for endoplasmic reticulum (ER) was showed colocalization with bacteria in some regions, however, no changes in the organelle distribution in the cytoplasm was observed (Figure 4). App also were observed around the nucleus forming clumps, and was detected in round zones similar to vesicles. On the contrary, Golgi Apparatus' distribution was affected with the App internalization in the endothelial cell, showing a perinuclear distribution (Supplementary Figure 1).

Interestingly, an elongation of the nuclei of infected cells was observed in all trial samples. The average length of nuclei at control samples was 14277.063 nm ± 21.85 nm, whilst for the endothelial cells interacted with App 4074 was 18344.528 ± 11.66 nm, 17389.621 ± 16.61 nm for JL03, 18324.956 ± 13.84 nm when L20 was internalized and 18109.861 nm ± 17.41 in the presence of AP76. The differences in lengths of control nuclei compared with the problem samples was statistically significant.

#### Vacuole Presence Evaluation by Acidic Organelles Labeling

As is shown in Supplementary Figure 3, the Lysotracker labeling results demonstrated that when the endothelial cells were infected with App an increased quantity of acidic vesicles (lysosomes) were produced in comparison with the control. This was observed for App JL03 and App L20, this result was similar to the effect that *E. coli* Bl21 (pUCmT::Vat) produced. On the contrary, no effect was observed for App 4074 neither for AP76.

#### *A. pleuropneumoniae* Serine Protease Expression Analysis

Westernblot results (Supplementary Figure 2) showed the recognition of a protein with *E. coli* vacuolizing toxin Vat antibody, in *A. pleuropneumoniae*. All App strains presented a 100 kDa band and other two bands of ~50 and 33 kDa. Negative control (*E. coli* Bl21 empty vector) only shows one band at 37 kDa whilst positive control *E. coli* Bl21 (pUCmT::Vat) show several bands including the ones present in App.

## Discussion

Endothelium is important in the pathogenesis of many bacteria. Based in a previous unpublished work carried out by this group, endothelium was one of the tissues that showed greater damage in post-mortem lung samples of pigs with swine pleuropneumonia, a finding in concordance with App's proven citotoxicity to endothelial cells (52). Therefore, studying App closely in relation with the endothelium could help better understand its pathogenesis.

As shown in the results obtained here, App strain 4074 had the higher adherence results, simiar to the results obtained for epithelial cells by Auger et al. (6). App JL03 and AP76 strains showed a linear behavior where adhesion increases over time, however the adhesion levels of App 4074 and L20 vary. Perhaps these observations are explainded by a weak affinity of these strains for the endothelium (53), but also due to the nature of endothelial cells, the continue process of pinocytosis that present, bacteria can enter and exit of the cell constantly, as is observed in other more distant bacteria as *E. coli* and *Shigella flexneri* (54).

As is known, App can remain in other tissues and organs of asymptomatic pigs, such as tonsils (55). Rare occurrence of App isolated from organs outside the respiratory system without causing sepsis (26, 36), led to analyze if it has the ability to invade host cells and spread to other organs using the endothelium to reach the bloodstream due to its intrinsic relationship as a material exchange barrier and involvement in the pathogenesis of several intracellular pathogens (28, 29, 56–58).

Internalization assay demonstrated low invasion rates for 4074, JL03, L20, and AP76 App strains, suggesting that few CFUs are necessary to cause the disease, however, this may be due to usage of its various virulence factors to infect and persist within the host and resorts to its invasion capacity in a lesser way (18). In a similar work *Haemophilus parasuis* also presented low invasion to aorta endothelial cells, nevertheless this is attributed by the authors due to possible low tropism to PBMEC cell line and this not being an optimal model for this particular bacteria (59, 60).

S4074 holds the lowest invasion percentage but highest adherence. Serotype 3 is considered a low virulence serotype and does not have high adhesion levels to EC, however shows greater adhesion than serotypes 5b and 7 and the highest invasion rate of all the 4 strains used in this work; evidencing differences in virulence and infection mechanisms between the serotypes.

It is worth to mention that when the interaction was made for 4 h, a considerable amount of detached and rounded EC was observed, mainly in the presence of serotype 5b (L20), followed by serotype 3 (JL03) due to App virulence and endothelial cytotoxicity (52). This results was less observed for serotypes 1 (4074) and 7 (AP76).

Invasion of non-phagocytic cells through rearrangement of the actin cytoskeleton is a common immune evasion mechanism used by most intracellular bacteria. Also, some pathogens modulate host microtubules and endocytic route as invasion mechanism, indicating that this organelle has a fundamental role in the infection process (20, 61).

As mentioned, in many cases the bacteria capable to invade cause a stimulus to the host that generates a rearrangement of the cytoskeleton resulting in the engulfing of the bacteria by non-phagocytic host cells. Therefore, once the internalization capacity of App was checked by CFU (6, 18, 38, 62), the experiments performed to analyze the possible involvement of the actin and tubulin cytoskeleton showed that even when an interaction of the bacterium with both kind of cytoskeleton was observed through co-localization, there was no visible rearrangement of any of them actin or tubulin, after App internalization. In addition at the time of using drugs capable of inhibiting the polymerization of these and therefore losing their function, the bacteria continued appearing inside of endothelial cells, showing that neither actin or tubulin cytoskeleton are necessary for App internalization. The results obtained shows remarkable differences with *Haemophilus parasuis, A. suis* and *Streptococcus suis*, whose invasion capacity is greatly diminished when a pretreatment of cytochalasin D is made, demonstrating an actin dependent internalization. On other hand, these differences with App are clear in comparison with *H. parasuis* and *A. suis* whose invasion requires also microtubules (53, 59, 60, 63).

The results show all 4 strains scattered inside the host and around to the perinuclear region forming clumps, this behavior is observed in other intracellular bacteria such as *Bartonella henselae* when invading human endothelial cells and in *Burkholderia* organism invading respiratory epithelium being accredited the phenomenon to microtubular trafficking (64, 65) the reason some intracellular bacteria use clumps rearrangement is currently unknown.

The tubular endolysosomal network is a system is in charge of bringing internalized molecules to their intracellular destination and is involved in nutrition processes, various cellular functions and maintaining cellular homeostasis. Due to their importance, opportunistic pathogens highjack these protein sorting pathways which allows them to internalize and replicate within the host cell avoiding degradation. In some cases, bacteria reside within vacuoles producing proteins that are transported by these sorting pathways, interfering with the function of endogenous regulators and endosomal substance trafficking systems (66).

The group A *Streptococcus* use LC3-associated phagocytosis (LAP) and enter the cell through vacuoles. After internalization pore-forming toxins damage the endosomal membrane allowing bacteria to escape into the cytoplasm where they induce reactive oxygen species (ROS), LAP formation and blocks ubiquitination inhibiting autophagy maintaining a neutral pH inside the autophagosome structure allowing cell proliferation (67). It is suspected a similar mechanism in App due to the round zones similar to vesicles observed in Figures 2, 3, showing bacteria inside them and at the circumference of the aforementioned structures, which indicate a possible form of internalization of the bacteria and transport method inside the host. App capacity to form lysosomes inside EC was analyzed. After 3 h interaction an increase of acidic organelles presence is seen in the case of App JL03 and L20 whereas for strains 4074 and AP76 shows basal lysosomal function, compared to negative control (EC without bacteria) implying a possible vacuolization function. Results where compared using as positive control *E. coli* Bl21 pUCmT::Vat a mutant that over expresses the characterized vacuolizing toxin Vat which induces vacuole formation and changes in permeability in epithelial cells (49). The increased number of acidic organelles in EC interacted with App imply the vacuolizing function for App leading to think a probable protein similar to Vat, present in App.

Polyclonal antibody against Vat SPATE toxins shows affinity to App protein extract indicating recognition to conserved epitopes of SPATEs (51). Westernblot results showed a band at approximately 100 kDa presumably App Subtisilin-like autotransporter protein AasP (104 kDa) that has homology with *E. coli* autotransporter proteins' binding site (68). Autotransporter proteins containing a serine protease motive are in volved in diverse functions like adhesion, colonization, cell host invasion, biofilm formation, and toxicity. AasP protein universally expressed in App is autocatalytic and has a maturation protease function. It shows homology to surface-localized autotransporters from various bacterial pathogens including *Mannheimia haemolytica, Bordetella pertussis*, and *Neisseria meningitidis*; suggesting a possible role in adhesion, colonization, internalization, and persistence in App (68, 69) although recombinant AasP antigen did not affected App virulence and did not protected pigs from App's colonization and infection (70). The other two bands recognized by anti-Vat antibody possibly indicate expression of more than one serine protease autotransporter or the presence of OmlA wich is maturated and released in the presence of AasP and is seen in SDS-PAGE gels as band of 50 kDa and a band of 33 kDa (69). Whether Aasp is responsible of inducing vacuole formation and has an active role in App invasion is yet to be determined.

Even when the true nature of the aforementioned intracytoplasmic structures similar to vesicles is unknown, they confirm the presence of App within the endothelial cytoplasm. Further investigation should be performed in order to elucidate the true nature of these structures.

Since intracellular pathogens exploit host cell signaling pathways to facilitate their uptake and survival within host cells (71, 72) and cytoskeleton does not seem to have a key role in App internalization; the possibility of bacteria entering the host through some other type of mechanism was analyzed. Therefore, the involvement of other cellular organelles with substance transport function was studied.

In cases such as reported for *Salmonellae* bacteria reach the perinuclear area an interacts with the host signaling pathways in order to reach the Golgi Apparatus where the intracellular bacterial replication begins (73), in *Bordetella bacilliformis* case if the Golgi Apparatus is disrupted invasion in highly diminished (64) showing a very important role in these bacteria invasion mechanisms. As seen in Figure 4 App continues to be observed within the endothelial cell. Golgi Apparatus normal distribution is altered and can be seen located in a predominant perinuclear area. Endoplasmic reticulum distribution is not disturbed by the presence of any of the 4 App strains, however we can find co-localization in some regions indicating a possible interaction between the organelle and the bacteria. ER is used by bacteria as a niche inside the host cell that ensures biosynthesis of nutrients such as lipids, carbohydrates and proteins and a safe place ideal to niche intracellularly (74). Whether direct or indirectly, an affection to these organelles is generated in the presence of intracellular App. How App interacts and relates to cellular organelles is yet to be known.

Figures 2–4 show in many areas enlarged nuclei on the endothelial cells when interaction with App occurs, this phenomenon has been previously reported in PAMs apoptosis induced by App (75), suggesting that the bacterium is interacting and causing morphological changes in them.

Many *Pasteurellaceae* organisms are identified as invasive pathogens but machanisms involved are not fully understood. With this work we state that *Actinobacillus pleuropneumoniae* is able to internalize in endothelial cells and could possibly be considered as an intracellular pathogen.

Although App can be found inside the cell and making apparent interactions with various organelles it is still unknown the specific route or mechanism it uses to achieve invasion. It is worth mentioning strains JL03 and L20 (serotypes 3 and 5b) show greater virulence against EC by presenting the highest adherence and internalization percentages and greater presence of acidic organelles after infection.

App might possess virulence factors enabling to achieve invasion through autophagy induction and transport through vesicles or it might benefit from cellular molecule exchange mechanisms, such as pinocytosis and caveolins, as an in and out way to host cell (21).

Further experiments must be performed in order to comprehend and elucidate *Actinobacillus pleuropneumoniae* invasion mechanism and test intracellular survival. Fully understanding of its pathogenesis will help developed better measures and diagnostic tools to prevent outbreaks of *A. pleuropneumoniae*.

## Data Availability Statement

The raw data supporting the conclusions of this article will be made available by the authors, without undue reservation.

## Author Contributions

BP-M and AG-B conceived the main research idea and designed the methodology. Experiments where performed by BP-M with support from AM-F. MD, FA-G, and MJ helped with the statistical analysis of the data. AG-B supervised the project. BP-M took the lead in writing the manuscript. All authors provided critical feedback and helped shape the research, analysis, and manuscript.

## Conflict of Interest

The authors declare that the research was conducted in the absence of any commercial or financial relationships that could be construed as a potential conflict of interest.

## Supplementary Material

Supplementary Figure 1 Actinobacillus pleuropneumoniae internalization effect on Golgi Apparatus. **(A)** Golgi Apparatus (red) normal distribution in swine endothelial cell, **(B–E)** Golgi Apparatus distribution is altered after App entry, migrating into a perinuclear position.

Supplementary Figure 2 Recognition of App total proteins with antibody anti- vacuolizing toxin Vat (100 kDa). (1) Molecular weight marker, (2) *E. coli* Bl21 empty vector total proteins (negative control), (3) *E. coli* Bl21 pUCmT::Vat total proteins (positive control), (4) App 4074 total proteins, (5) App JL03 total proteins (6) App L20 total proteins, (7) App AP76 total proteins. All App samples show a band at 100 kDa, one between 52 and 37 kDa and another one between 37 and 25 kDa. Positive control shows same bands as App whilst negative control only has one band at 37 kDa.

Supplementary Figure 3 Acidic organelles formation after internalization assay. **(A)** Endothelial cells without bacteria, **(B)** Endothelial cells + *E. coli* Bl21 pUCmT::Vat, **(C)** Endothelial cells + App 4074, **(D)** Endothelial cells + App JL03, **(E)** Endothelial cells + App L20, **(F)** Endothelial cells + App AP76. Endothelial cells interacted with each bacterial strain for 3 h followed by labeling for 30 min with Lysotracker Deep Red [75 nm]. A remarkable increased of acidic organelles is seen when interacted with *E. coli* and with App JL03 and L20 but no notable difference between negative control and cells interacted with App 4074 and AP76.
