# Supplementary figures and images for: Actinobacillus pleuropneumoniae Interaction With Swine Endothelial Cells
Source: Front Vet Sci. 2020 Oct 29;7:569370. doi: 10.3389/fvets.2020.569370 (PMC7658479; doi:10.3389/fvets.2020.569370)

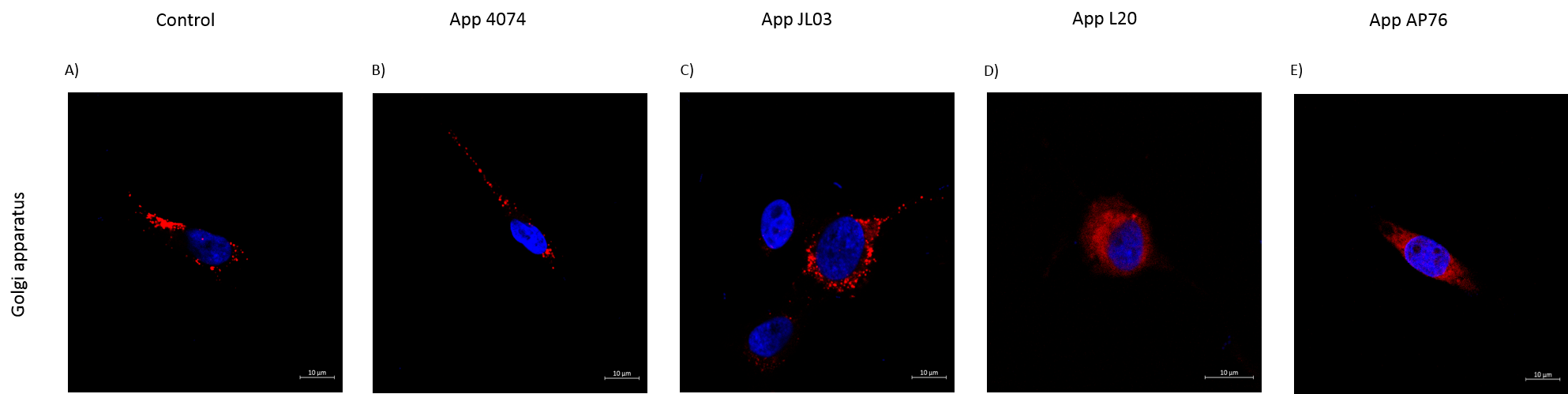

Supplement: Supplementary Figure 1 — Actinobacillus pleuropneumoniae internalization effect on Golgi Apparatus. (A) Golgi Apparatus (red) normal distribution in swine endothelial cell, (B–E) Golgi Apparatus distribution is altered after App entry, migrating into a perinuclear position. [file Image_1.TIF]

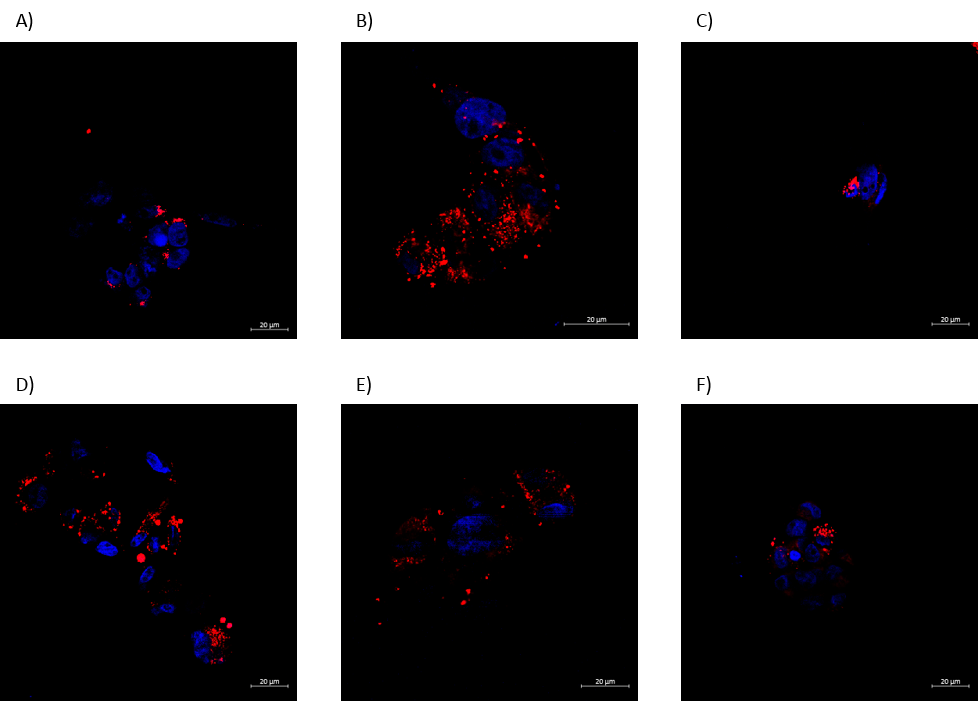

Supplement: Supplementary Figure 3 — Acidic organelles formation after internalization assay. (A) Endothelial cells without bacteria, (B) Endothelial cells + E. coli Bl21 pUCmT::Vat, (C) Endothelial cells + App 4074, (D) Endothelial cells + App JL03, (E) Endothelial cells + App L20, (F) Endothelial cells + App AP76. Endothelial cells interacted with each bacterial strain for 3 h followed by labeling for 30 min with Lysotracker Deep Red [75 nm]. A remarkable increased of acidic organelles is seen when interacted with E. coli and with App JL03 and L20 but no notable difference between negative control and cells interacted with App 4074 and AP76. [file Image_3.TIF]
